# *Toxoplasma gondii* pseudocyst in a transbronchial biopsy: a case report

**DOI:** 10.1186/s13256-016-1039-8

**Published:** 2016-09-22

**Authors:** Caio César Inaco Cirino, André Peluso Nogueira, André Amate Neto, Patricia Cristina Urbano, Tales Rubens de Nadai

**Affiliations:** 1 Americo Brasiliense State Hospital, 1260 Alameda Aldo Lupo, 14820-000 Américo Brasiliense, SP Brazil; 2 Department of Surgery and Anatomy, Ribeirão Preto School of Medicine, University of São Paulo, 3900 Avenida Bandeirantes, 14048-900 Ribeirão Preto, SP Brazil

**Keywords:** Bronchoscopy, Toxoplasmosis, *Toxoplasma gondii*, Transbronchial biopsy, Appendicitis

## Abstract

**Background:**

We herein present a case in which a *Toxoplasma* cyst was found in a transbronchial biopsy specimen from an immunocompetent patient with negative serology for the parasite.

**Case presentation:**

An 18-year-old Brazilian man presented with a 1-week history of dyspnea and fever and was diagnosed with right lower lobe pneumonia. He began inpatient treatment with intravenous antibiotics. During treatment, a bronchoscopy with bronchoalveolar lavage and transbronchial biopsy was performed. Anatomopathological examination of the transbronchial biopsy showed a small fragment of lung parenchyma with discrete septal thickening and a rounded structure, suggestive of a pseudocyst containing *Toxoplasma gondii* bradyzoites. However, serological tests were negative for immunoglobulin G and immunoglobulin M.

**Conclusions:**

Bronchoscopy is a minimally invasive, effective diagnostic and therapeutic method. Despite the fact that the *Toxoplasma* pseudocyst in the present case was not the cause of the patient’s comorbidities, bronchoscopy with transbronchial biopsy allowed for an incidental diagnosis of a *Toxoplasma* pseudocyst with minimal invasiveness.

## Background

Toxoplasmosis is a disease that affects mammals and birds worldwide and is caused by the parasite *Toxoplasma gondii*. It can take several forms in humans, ranging from oligosymptomatic to severe. Symptoms mainly involve the heart, liver, lungs, eyes, and brain, and the most serious cases are diagnosed in patients with impaired immunity. We herein present a case in which a single *Toxoplasma* pseudocyst was found in a transbronchial biopsy specimen from an immunocompetent patient with negative serology for the parasite.

## Case presentation

An 18-year-old Brazilian man developed dyspnea and fever of one week’s duration and was then diagnosed with right lower lobe pneumonia. Before this incident, he had not experienced any medical complications, lived in full sanitary conditions, denied previous symptoms related to disease, did not have any pets, and denied eating raw meat. He then began hospital inpatient treatment with intravenous antibiotics, and third-generation cephalosporin (Ceftriaxone) and a macrolide (Clarithromycin) were prescribed for 8 days. Laboratory tests from the patient’s admittance showed mild anemia with a hemoglobin (Hb) level of 11.7 g/dL, with normal mean corpuscular volume (MCV) and mean corpuscular hemoglobin (MCH). Other biochemical results were as follows: creatinine 0.9 mg/dL, urea 32 mg/dL, total bilirubin 0.2 mg/dL, glucose 90 mg/dL, sodium 134 mEq/L, potassium 3.7 mEq/L, and C-reactive protein 138.2 mg/L. Leucocytes levels were a little low, with 2700 cells/μL. Blood and urine cultures were negative for infection and serological tests for human immunodeficiency virus (HIV), hepatitis B and C, and syphilis were all negative.

**Fig. 1:**
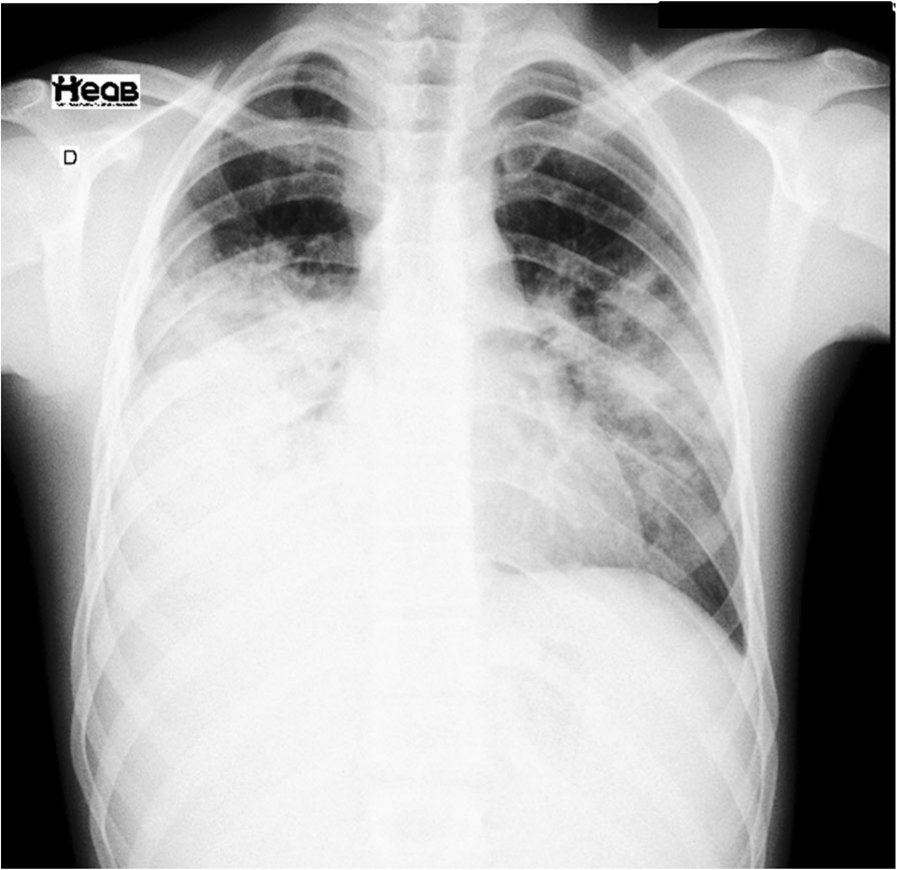
Chest X-ray on the day of hospitalization. There was a heterogeneous opacity in the middle and lower third of the right lung field and in the middle third of the left lung field

On the fourth day of this related treatment, our patient developed abdominal distension and pain in his lower right abdominal quadrant. Acute appendicitis was confirmed by ultrasound examination, and he underwent laparoscopic surgery the same day. Our patient was then taken to our intensive care unit for worsening of his clinical condition. On the fourth postoperative day, our patient developed fever associated with right pleural effusion, which was a lymphocytic exudate with an adenosine deaminase concentration of 17.99 U/L and negative culture (Fig. 1). Thus, the attending physicians requested a bronchoscopy with bronchoalveolar lavage and transbronchial biopsy. The transbronchial fluid had negative cultures for infection. At that time, the antibiotic spectrum was extended for treatment of the sepsis, including piperacillin/tazobactam for 10 days.

**Fig. 2:**
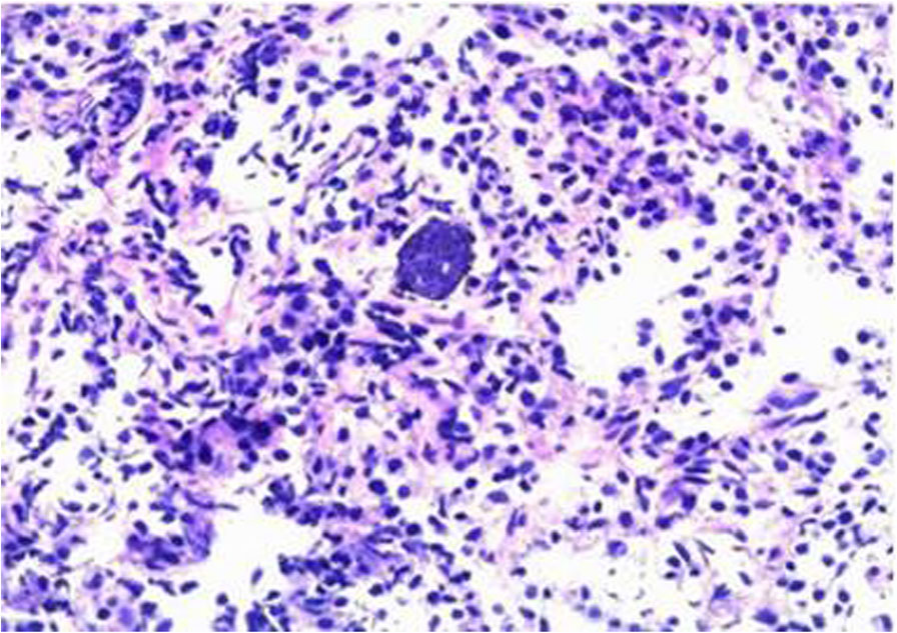
Anatomical pathology of the transbronchial biopsy. There was a small pulmonary parenchyma fragment and a rounded structure (pseudocyst) containing bradyzoites of *Toxoplasma gondii*

Anatomopathological examination of the transbronchial biopsy specimen showed a small fragment of lung parenchyma with discrete septal thickening and a rounded structure suggestive of a pseudocyst containing *Toxoplasma gondii* bradyzoites (Fig. 2).

**Fig. 3:**
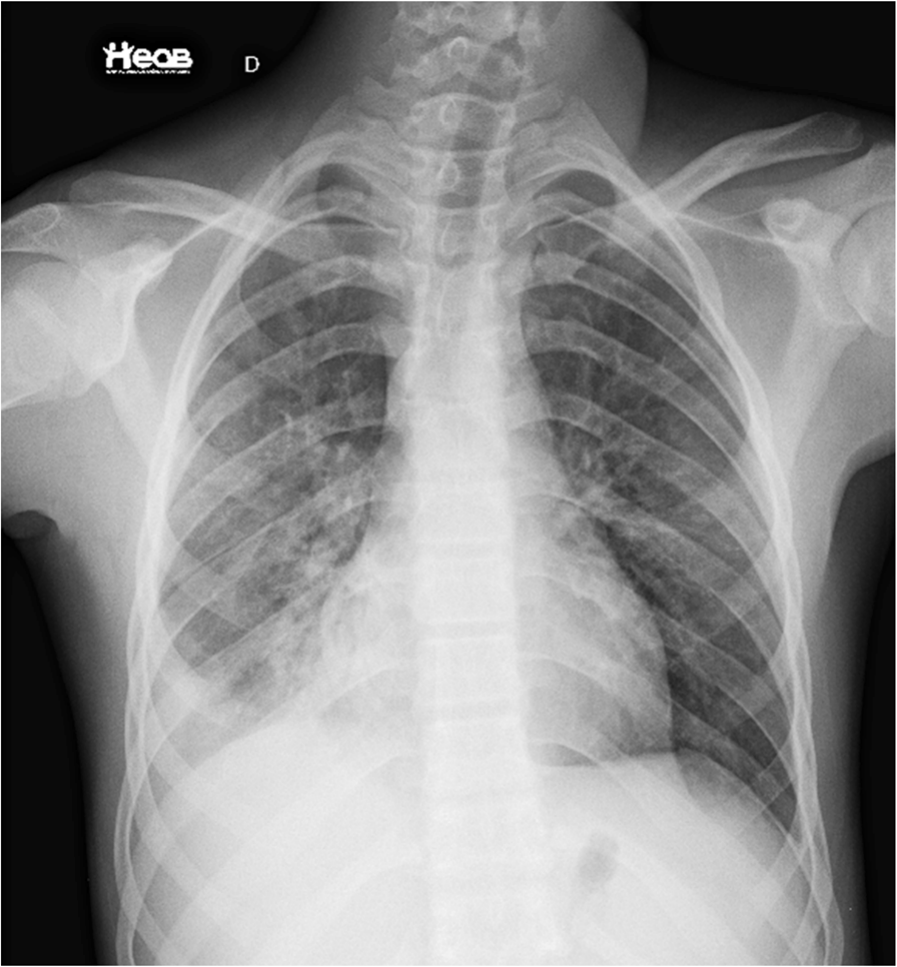
Chest X-ray on the day of discharge. There was a small heterogeneous opacity in the lower third of the right lung field

Our patient showed progressive improvement after antibiotic therapy with piperacillin/tazobactam, and was discharged on the 14th day after surgery. A chest radiograph showed significant improvement in lung opacification (Fig. 3) and serological tests for toxoplasmosis were negative for immunoglobulin G and immunoglobulin M.

Our patient was followed up on an outpatient basis for 2 years without any signs of recurrence of lung infections or other comorbidities. Serology for toxoplasmosis were repeated and remained negative.

## Discussion

*Toxoplasma gondii* is a protozoan [1] whose obligate intracellular life cycle of infection is divided into different stages: tachyzoite; merozoite, which gives rise to gametes and eggs; and zygote, which gives rise to the oocyst. In the external environment, the oocyst undergoes meiotic division and forms two sporocysts, each with four sporozoites that are highly resistant to disinfectants and can last up to 5 years in the environment [2, 3]. If a human is infected, the intestinal oocysts become numerous tachyzoites through asexual reproduction. The human being is considered an intermediate host because final sexual maturation occurs only in the intestines of dogs [4].

Tachyzoites cause acute infection in which some parasites become numerous cysts containing bradyzoites, as in the present case. This occurs after the immune response that controls the infection is activated; a positive result on serological tests is then obtained [5].

During chronic infection, bradyzoites slowly multiply within their cysts, and multiplication may even stop. This multiplication occurs within the cytoplasmic vacuoles, whose membranes become the cyst capsule. The capsule, composed of glycoproteins, isolates bradyzoites from the immune mechanisms of the host. Thus, the cysts persist intact for months to years and can result in negative serological tests.

The primary *T. gondii* infection pathway involves fecal–oral transmission or eating meat from other intermediate hosts, such as pigs, poultry, sheep, and cattle. *T. gondii* infection can also be transmitted to the fetus through the placenta. Eosinophilia is generally an atypical finding during infection with the protozoan. Clinical manifestations are complicated and atypical, and most infections are latent or acute; differential diagnosis is mainly based on direct detection of *Toxoplasma* tachyzoites or serological techniques [6].

Immunocompetent patients with acute *T. gondii* infection are usually asymptomatic. However, up to 10 % of cases can have nonspecific and self-limited clinical manifestations that rarely require treatment. Lymphadenopathy (bilateral, symmetrical, and hardened) is one of the most common clinical manifestations. Pulmonary manifestations in immunocompetent patients are rare and can appear as dry cough, fever, nonspecific symptoms (such as myalgia, weakness, and rash) and radiological findings that can also correspond to the findings of atypical pneumonia (diffuse interstitial pulmonary infiltrates) [7].

The unprecedented finding in the present case was a pseudocyst with bradyzoites in the lung parenchyma in a patient with negative serology for the parasite. We found no descriptions of similar cases in the literature, thus making our experience in this case noteworthy. This finding was likely associated with direct infection by inhalation of a cyst that was not activated in the lung environment. More remarkably, we were able to extract this cyst from the transbronchial biopsy specimen. Diagnosis of acute lung infection with a protozoan at the time of biopsy is truly akin to finding a needle in a haystack.

## Conclusions

*T. gondii* infection has various clinical presentations and multiple forms. Humans are intermediate hosts of *T. gondii* because we do not provide a favorable environment for the parasite to complete its sexual cycle. Bronchoscopy is a minimally invasive, effective diagnostic and therapeutic method. In this case, despite the fact that the *Toxoplasma* cyst was not the cause of our patient’s comorbidities, bronchoscopy with transbronchial biopsy allowed for an incidental diagnosis of a *Toxoplasma* pseudocyst with minimal invasiveness.
